# Decreased daily exercise since the COVID-19 pandemic and the deterioration of health-related quality of life in the elderly population: a population-based cross-sectional study

**DOI:** 10.1186/s12877-022-03316-9

**Published:** 2022-08-16

**Authors:** Koji Tamai, Hidetomi Terai, Shinji Takahashi, Hiroshi Katsuda, Nagakazu Shimada, Hasibullah Habibi, Hiroaki Nakamura

**Affiliations:** 1 Department of Orthopaedic Surgery, Osaka Metropolitan University Graduate School of Medicine, 1‑5‑7, Asahimachi, Abenoku, Osaka City, Osaka 545-8585 Japan; 2 Department of Orthopaedic Surgery, Shimada Hospital, 100-1, Kusiyama, Habikino, Osaka 583-0875 Japan

**Keywords:** COVID-19, Health-related quality of life, Residents, Exercise, Physical activity

## Abstract

**Backgrounds:**

The current prolonging state of the coronavirus disease (COVID-19), could affect many aspects of people’s lives, especially the elderly population who experience a decrease in regular exercise. However, whether this decrease in regular exercise affects health-related quality of life (HRQOL) of the elderly population, remains unclear.

**Methods:**

The current population-based cross-sectional survey aimed to identify the relationship between the decrease in regular exercise since the COVID-19 pandemic and any changes in the HRQOL in the general elderly Japanese population. This study was conducted as a part of the COVID-19 vaccination program in Habikino city in Japan, between June and July 2021 using printed questionnaires. The participants included residents of the city who were aged ≥ 65 years, and were being vaccinated for COVID-19 at the city’s center. The EuroQoL 5-dimension 5-level (EQ-5D-5L) was assessed at two different time points (pre-pandemic and current). Data on lifestyle changes, including their regular exercise routine since the pandemic, were collected.

**Results:**

Finally, 14,494 participants (45.3% of the city’s total elderly residents) were enrolled. Among them, 4321 participants (29.8%) had experienced a decrease in regular exercise since the pandemic. These participants showed a significantly higher rate of deterioration in all the EQ-5D-5L domains than the participants who did not experience a decrease in regular exercise. In the multivariate logistic regression analysis, participants with a decrease in regular exercise were significantly related to the EQ-5D-5L index deterioration compared to those with an unchanged regular exercise routine (*p* < 0.001, adjusted odds ratio = 5.60) independent of age, sex, body mass index (BMI), and the existence of back pain, joint pain, and/or numbness of extremities.

**Conclusion:**

The current survey that included 45% of the elderly people living in a city revealed that up to 30% of them had experienced a decrease in the regular exercise since the COVID-19 pandemic. This decrease was significantly related to HRQOL deterioration independent of age, sex, BMI, baseline activities of daily living status, and musculoskeletal symptoms. Our data could be useful for understanding the current problem and provide a strong basis for the creation of exercise guidelines for the post-COVID-19 era.

## Background

The coronavirus disease 2019 (COVID-19) pandemic has had many effects on the lives and health of people worldwide [1]. Severe restrictions on daily life, including home confinement or lockdown, were partially effective in preventing the spread of COVID-19 in the community. However, a prolonged period with them could also negatively affect many aspects of humans’ lives, including a decrease in regular exercise [2, 3]. Therefore, the importance of maintaining one’s physical activity was highlighted as an important strategy at the beginning of the pandemic [4, 5]. However, evidence demonstrated that the total physical activity time in the elderly population decreased by approximately 35% after the COVID-19 pandemic [3].

Regular physical activity and/or exercise, play an important role in preventing adverse health outcomes [6, 7]. Furthermore, studies have demonstrated that regular exercise is a vital management strategy for a better health-related quality of life (HRQOL), especially in the elderly population [2, 8, 9]. However, how the decrease in regular exercise since the COVID-19 pandemic affects the HRQOL of the general elderly population remains unclear.

An online survey is the current standard study conductance method. However, the method could not adequately evaluate the all-inclusive situation of the elderly population, as only the ones who were able to use internet-connected devices and who were actively registered with a survey by themselves, could be evaluated [3]. This would result in a sampling bias. Therefore we used printed questionnaires and distributed them manually instead.

## Patients and methods

### Aim

The current population-based cross-sectional survey using self-administered, printed questionnaires aimed to identify the relationship between the decrease in regular exercise since the COVID-19 pandemic and the change in HRQOL in the general elderly population.

### Ethical approval

All study participants provided written informed consent. The study protocol was approved by the Institutional Review Board (approval No: 2021–110). The official approval for the current survey was also obtained from the mayor of Habikino city. All information has been handled in accordance with the standards for privacy of individually identifiable health information of health insurance portability and accountability act.

### Survey design

The survey was conducted in accordance with the COVID-19 vaccination program of Habikino City, Osaka, Japan, between June and July 2021. In the Japanese COVID-19 vaccination system, the elderly population aged ≥ 65 years got vaccinated for COVID-19 on a priority basis, and each city established its own vaccination program for residents. Habikino city is a mid-sized suburban city located in Eastern Osaka Prefecture, Japan. The city has a total population of approximately 110,106 and an elderly population aged ≥ 65 years of 33,191 as of February 2021 [10]. In the city’s vaccination program, elderly residents collected in one vaccination center for COVID-19 vaccination between June and July 2021. After obtaining permission from the city and the approval of the IRB of our institution, we invited all elderly residents who received their vaccination at the center to participate in our survey. Participants who provided informed consent were enrolled in this study. Participants could withdraw anytime they wished, during or after completing the questionnaire.

### Questionnaire

All information were collected by one survey conducted from June to July, 2021. Within the questionnaires distributed to every participant, some questions were about the pre-pandemic status, some about the current status and some about the both of them. They were instructed to refer to the following two time periods when completing such questionnaire: “pre-pandemic” (March to April, 2020) and “current situation” (June to July, 2021).

#### General information

Questions included the participant’s age, sex, body height, body weight, and musculoskeletal symptoms including back pain, joint pain (hip, knee, and/or ankle joint), and numbness (upper, and/or lower extremities). In addition, changes in exercise habits (unchanged, increased, decreased, no exercise habit) since the COVID-19 pandemic were recorded.

#### Activities of daily living (ADLs)

The criteria for determining the participants’ level of independence in performing ADLs were based on the definition provided by the Japanese Ministry of Health, Labour, and Welfare [11]. The survey asked the participants to answer questions regarding ADL during the pre-pandemic period. Grade J included individuals who had some sort of disability but was almost independent while performing daily life activities and could get out of the house by themselves. Grade A included individuals who are almost independent in performing indoor daily life activities but cannot go outside without assistance. Grade B included individuals who required some assistance in performing indoor daily life activities and could be out of the bed most of the time and maintain a sitting position. Grade C included those individuals who stayed in bed all the time and required assistance for toileting, eating, and changing clothes. Patients were divided int two groups; Grade J or Grade A, B, and C.

#### HRQOL index

Each participant’s HRQOL at the two time points (pre-pandemic and current) was assessed using the EuroQoL 5-dimension 5-level (EQ-5D-5L) in this cross-sectional survey [12]. The EQ-5D-5L measures HRQOL consisting of five severity levels from 1-to-5 for five domains: mobility, self-care, usual activities, pain/discomfort, and anxiety/depression. The domain scores were then converted into a summarized index according to the published values proposed in previously reported tables. After conversion, the index was between 0 and 1.0 (minimum score: 0, full score:1.0) [13, 14].

### Statistical analysis

All participants were divided into four groups according to the changes in exercise habit (unchanged, increased, decreased, no exercise habit). Age, sex, height, weight, body mass index (BMI), musculoskeletal symptoms at baseline, and ADL at baseline were compared among the four groups using one-way analysis of variance (ANOVA) for continuous variables and the Chi-squared test for categorical variables. As a post-hoc analysis of the chi-squared test, residual analysis was performed. The result of the residual analysis was described as *p* < 0.05 when standardized residual values were > 1.96, according to the Haberman method [15]. The number of individuals whose EQ-5D-5L domains deteriorated one or more levels during the pandemic compared to the pre-pandemic situation were compared among the four groups using Chi-squared test with residual analysis. The average score of converted EQ-5D-5L index was compared between two groups using unpaired t test, and among the four groups using one-way ANOVA with the Tukey test as post-hoc analysis. As EQ-5D-5L index is the primary outcome of this current study, we also calculated the power of our analysis. Finally, variables with a significance level of *p* < 0.05, in the univariate analysis, and regular exercise were included in the multivariable logistic regression model as explanatory variables. In this analysis, the EQ-5D-5L index that decreased for > 0.1 point was set as an objective variable [16]. The adjusted odds ratios (aORs) and 95% confidence intervals of the dependent variables were calculated. Further, the interaction between regular exercise and other explanatory variables was calculated in the analysis. All analyses were performed using IBM SPSS software (version 23.0; IBM Corp., Armonk, NY, USA). Statistical significance was set at *p* < 0.05 and continuous variables were demonstrated with average ± 1.0 standard deviation.

## Results

### Number of participants

**Fig. 1:**
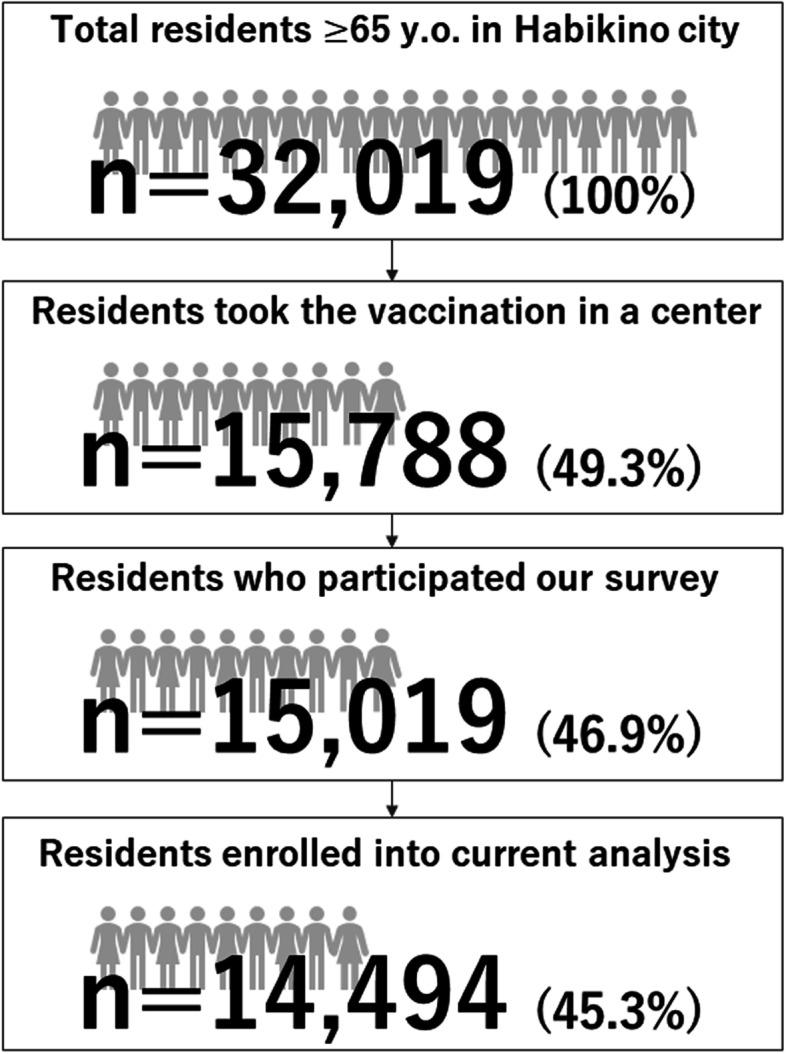
Study participants. y.o.: years old

Overall, 15,788 elderly residents were vaccinated at the center during the examination period. Among them, 15,019 residents agreed to participate in our survey. After collecting the data, a total of 525 participants were excluded from the analysis due to the lack of necessary information, such as age (*n* = 79), sex (*n* = 314), body height/weight (*n* = 132), and changes in exercise habit (*n* = 270). Finally, 14,494 participants (45.3% of the total elderly residents of Habikino City) were included in the current analysis (Fig. 1).

### Grouping based on changes in exercise habit

Among the 14,494 participants, 8410 participants (58.1%) answered “unchanged” when asked about their exercise habit since the pandemic, while 483 participants (3.3%) answered “increased”, and 4321 participants (29.8%) answered “decreased”. Additionally, 1280 participants (8.8%) answered “no exercise”, indicating that they did not do any regular exercise even before the pandemic.

### Univariate comparisons among the groups

The distribution of age, sex ratio, height, weight, BMI, number of individuals with symptoms, and ability to perform ADL showed significant differences among the four groups (*p* < 0.001, Table 1). Residual analysis revealed that the unchanged group were more likely to be 70–80 years old, men and had a lower number of individuals with back, or joint pain, or numbness (*p* < 0.05, respectively). On the other hand, the decreased group were more likely to be 65–70 years of age, women and had a higher number of individuals with back or joint pain, or numbness (*p* < 0.05).

**Table 1:** Demographics of the four groups classified by the change in exercise habit

	Overall	Unchanged	Decreased	Increased	No exercise habit	*p*-value
Numbers	14,494	8410 (58.1)	4321 (29.8)	483 (3.3)	1280 (8.8)	
Age						< 0.001^†^
65–70	4902 (33.8)	2677 (31.8)*	1576 (36.5)*	202 (41.9)*	447 (34.9)	
71–75	4224 (29.1)	2426 (28.8)	1291 (29.9)	134 (27.7)	373 (29.1)	
76–80	2802 (19.3)	1720 (20.5)*	801 (18.5)	79 (16.4)	202 (15.8)*	
81–85	1599 (11.0)	992 (11.8)*	421 (9.7)*	50 (10.4)	136 (10.6)	
86-	967 (6.7)	595 (7.1)*	232 (5.4)*	18 (3.7)*	122 (9.5)*	
Sex						< 0.001^†^
Male	6665 (46.0)	4416 (52.5)*	1520 (35.2)*	230 (47.6)	499 (39.0)*	
Female	7829 (54.0)	3994 (47.5)*	2801 (64.8)*	253 (52.4)	781 (61.0)*	
Height (cm)	158.9 ± 8.9	159.7 ± 9.0	157.8 ± 8.6	159.4 ± 8.6	157.5 ± 9.0	< 0.001^#^
Weight (kg)	58.2 ± 11.0	58.5 ± 11.0	57.3 ± 10.8	59.0 ± 10.9	58.1 ± 11.9	< 0.001^#^
BMI	22.9 ± 3.4	22.8 ± 3.3	22.9 ± 3.3	23.1 ± 3.3	23.3 ± 4.0	< 0.001^#^
Symptoms						
Back pain	3462 (23.9)	1657 (19.7)*	1277 (29.6)*	149 (30.8)*	379 (29.6)*	< 0.001^†^
Joint pain	1291 (8.9)	546 (6.5)*	531 (12.3)*	51 (10.6)	163 (12.7)*	< 0.001^†^
Numbness	708 (4.9)	322 (3.8)*	248 (5.7)*	37 (7.7)*	101 (7.9)*	< 0.001^†^
ADL						< 0.001^†^
Grade J	12,509 (86.3)	7200 (85.6)*	3910 (90.5)*	433 (89.6)*	966 (75.5)*	
Grade A, B, C	909 (6.3)	493 (5.9)*	209 (4.8)*	13 (2.7)*	194 (15.2)*	
Missing	1076 (7.4)	717 (8.5)	202 (4.7)	37 (7.7)	120 (9.4)	

Continuous variables were presented with an average ± 1.0 standard deviation. Figure in parentheses indicates the percentage. #One-way analysis of variance; †Chi-squared test; *p < 0.05, residual analysis; *BMI* body mass index, *ADL* Activity of daily living

### Comparison of EQ-5D-5L change among the groups

The number of participants whose EQ-5D-5L domain deteriorated were significantly different among the four groups (*p* < 0.001, respectively, Table 2). Residual analysis demonstrated that the number of participants with deterioration in all domains, including mobility, self-care, usual activities, pain/discomfort, and anxiety/depression, was higher in the decreased group than predicted outcomes (*p* < 0.05, respectively). In terms of the change in EQ-5D-5L index between pre- and during-pandemic in the overall cohort, it significantly decreased since the pandemic from 0.963 ± 0.09 to 0.954 ± 0.115 (*p* < 0.001, power analysis > 0.999). Comparing the four groups, the decreased group showed a significantly greater reduction compared to the unchanged group with a difference of 0.015, increased group with a difference of 0.015, and no exercise-habit group with a difference of 0.012 (*p* < 0.001, respectively, Table 3). The current study revealed significant differences in the degree of HRQOL index decrease between the population who had unchanged exercise habits and those who decreased their exercise routine (*p* < 0.001). While, there were no significant differences in the reduced EQ-5D-5L total index between the unchanged group and increased group, the unchanged group and no-exercise habit group, and increased group and no-exercise habit group (*p* = 0.996, 0.137 and 0.737 respectively.) The power of these analyses was > 0.999 respectively.

**Table 2:** The comparison of deterioration of EQ-5D-5L subdomains

	Unchanged	Decreased	Increased	No exercise habit	*p*-value
Mobility					< 0.001^†^
Stable	7452 (98.7)*	3777 (93.6)*	430 (97.3)	1114 (96.8)	
Deteriorated	96 (1.3)*	259 (6.4)*	12 (2.7)	37 (3.2)	
Self-care					< 0.001^†^
Stable	7503 (99.7)*	3953 (98.2)*	430 (97.5)*	1137 (99.0)	
Deteriorated	23 (0.3)*	73 (1.8)*	11 (2.5)*	12 (1.0)	
Usual activities					< 0.001^†^
Stable	7435 (99.1)*	3770 (94.4)*	435 (98.4)	1121 (98.2)	
Deteriorated	71 (0.9)*	224 (5.6)*	7 (1.6)	21 (1.8)	
Pain/discomfort					< 0.001^†^
Stable	7335 (99.0)*	3761 (95.4)*	430 (97.7)	1093 (98.1)	
Deteriorated	71 (1.0)*	181 (4.6)*	10 (2.3)	21 (1.9)	
Anxiety/depression					< 0.001^†^
Stable	7314 (98.8)*	3668 (93.1)*	428 (98.2)	1096 (98.0)*	
Deteriorated	90 (1.2)*	272 (6.9)*	8 (1.8)	22 (2.0)*	

Number in parentheses indicates the percentage

^†^: Chi-squared test, *: *p* < 0.05 in residual analysis, *EQ-5D-5L* EuroQol-5-dimension 5-level

**Table 3:** The change in EQ-5D-5L total index

	Unchanged (*n* = 8410)	Increased (*n* = 483)	Decreased (*n* = 4321)	No exercise habit (*n* = 1280)	*p*-value
Pre-pandemic	0.972 ± 0.09	0.973 ± 0.07	0.965 ± 0.09	0.922 ± 0.15	Overall: < 0.001 Post hoc: U-I: 0.991 U-D: 0.003 U-N: < 0.001 I-D: 0.360 I-N: < 0.001 D-N: < 0.001
Current	0.967 ± 0.10	0.968 ± 0.08	0.946 ± 0.12	0.918 ± 0.15	Overall: < 0.001 Post hoc: U-I: 1.000 U-D: < 0.001 U-N: < 0.001 I-D: < 0.001 I-N: < 0.001 D-N: < 0.001
Range of reduction	0.003 ± 0.025	0.003 ± 0.042	0.018 ± 0.063	0.006 ± 0.042	Overall: < 0.001 Post hoc: U-I: 0.996 U-D: < 0.001 U-N: 0.137 I-D: < 0.001 I-N: 0.737 D-N: < 0.001

Continuous variables were presented with an average ± 1.0 standard deviation. Overall p value: One-way Analysis of variance, Post hoc: Tukey test in each comparison, *EQ-5D-5L* EuroQol-5-dimension 5-level, *U* Unchanged group, *I* increased group, *D* decreased group, *N* no exercise habit group

### Multivariate regression model

Age, sex, lower BMI, and existence of back pain, joint pain, and/or numbness were independently related to EQ-5D-5L index deterioration (*p* < 0.001, 0.029, 0.004, 0.009, < 0.001. and 0.003, Table 4). In terms of the change in regular exercise, the participants with decrease in regular exercise showed a significant EQ-5D-5L total index deterioration compared to those with a unchanged regular exercise (*p* < 0.001, aOR = 5.60). There was no significant interaction between regular exercise and other explanatory variables in the logistic regression analysis (age * change in exercise habit: *p* = 0.246, sex * change in exercise habit: *p* = 0.976, BMI * change in exercise habit: *p* = 0.853, back pain * change in exercise habit: *p* = 0.471, joint pain * change in exercise habit: *p* = 0.252, numbness * change in exercise habit: *p* = 0.458, ADL * change in exercise habit: *p* = 1.000).

**Table 4:** Multivariable logistic regression model

Objective variable: individuals whose EQ-5D-5L total index decreased by > 0.1 point					
Explanatory variables		Reference	aOR	*p*-value	95%CI
Age	> 75	60 to 75	1.78	< 0.001	1.43–2.21
Sex	Female	Male	1.30	0.029	1.03–1.64
BMI	< 18	18 to 30	1.63	0.004	1.17–2.27
	> 30	18 to 30	1.59	0.086	0.94–2.71
Symptoms	Back pain	No back pain	1.37	0.009	1.08–1.73
	Joint pain	No joint pain	1.67	< 0.001	1.26–2.23
	Numbness	No numbness	1.69	0.003	1.19–2.41
ADL	A, B, C	J	1.22	0.309	0.83–1.70
Regular exercise	Increased	Unchanged	1.80	0.099	0.90–3.63
	Decreased	Unchanged	5.60	< 0.001	4.33–7.25
	No habit	Unchanged	1.77	0.014	1.12–2.78

*BMI* Body mass index, *ADL* activity of daily living, *aOR* adjusted odds ratio, *CI* confidence interval

## Discussion

The current population-based survey involving 45% of elderly residents of a suburban city in Japan revealed that up to 30% had experienced a decrease in the regular exercise since the COVID-19 pandemic. These elderly individuals showed significant HRQOL deterioration independent of age, sex, BMI, baseline ADL status, and musculoskeletal symptoms.

There is evidence supporting the health benefits of physical activity, and regular exercise is a critical factor within health promotion settings. For example, the volume of physical activity can lead to improved perceptions of health in adolescents [17]; there is a curvilinear relationship between physical activity and mortality in older adults [18], and routine physical activity is reported to be related to the reduction of the risk of multiple chronic medical conditions [19]. Therefore, many physical activity guidelines recommend 150 min/week of moderate-to-vigorous intensity physical activity [20, 21]. However, it was reported in another study that the total physical activity time per week for older adults decreased by 26.5% in average from January to April 2020, when the first wave of the COVID-19 pandemic in Japan occurred [3]. In accordance with this previous study, we found that up to 30% of the general elderly individuals recognized a decrease in their regular exercise since the COVID-19 pandemic.

Our primary hypothesis was that decrease in exercise after pandemic might affect the HRQOL negatively because of a deterioration of musculoskeletal symptoms such as back pain, joint pain and numbness. However, our multivariate analysis revealed that the decrease in exercise related to the decrease in HRQOL independently from musculoskeletal symptoms. Additionally, there was no interaction between musculoskeletal symptoms and change in exercise routines. All these findings indicated that there might be direct relationship between exercise and HRQOL, and potentially indicated the importance to encourage the elderly population to keep their exercise habit ongoing even during the COVID-10 pandemic.

In terms of the relationship between HRQOL and COVID-19, the pandemic itself has a negative impact on HRQOL. For example, Algahtani et al. reported that the COVID-19 pandemic has significantly influenced various aspects of individuals’ HRQOL, including their physical and psychological health [22]. The current study also demonstrated that the average HRQOL index of all participants had significantly decreased. However, the average degree of decrease was 0.008, which might be less than the meaningful value [16]. On the other hand, the current study revealed clear differences in the degree of HRQOL index decrease between the population who have unchanged exercise routine and those who decreased their exercise routine, with a difference of 0.015, which could be considered as a meaningful value [16]. In the analysis of subdomains of HRQOL, the population with decreased exercise habit demonstrated a significantly higher rate of deterioration in all five domains, including mobility, self-care, usual activities, pain, and anxiety compared to those with unchanged exercise habits. It might be difficult to identify causality in the relationship between excise reduction and deterioration in mobility, self-care, and usual activity subdomains in the current cross-sectional study. However, because there is enough evidence showing that exercise has a positive effect on mental health, including anxiety and depression, and body pains [23, 24], the decrease in exercise potentially causes deterioration in the subdomains of anxiety/depression and pain.

In terms of the importance of exercise in the elderly population, SPINE20 which is an international advocacy group, called to G20 leaders and published a recommendation for the G20 countries to adopt a strategy to promote daily physical activity and exercises among the elderly population to maintain an active and independent life with a healthy spine, particularly since COVID‑19 pandemic [25]. Our results indicated that regular exercise was decreased in 30% of the elderly population since the COVID-19 pandemic. Additionally, the results of current multivariate analysis revealed that exercise reduction in the elderly population was related to deterioration of HRQOL independent of age, sex, BMI, musculoskeletal symptoms, and ADL status. These results support a negative relationship between decrease in exercise routine and HRQOL, and could support SPINE20’s statement of the recommendations. Additionally, our results emphasize the importance of maintaining regular exercise during the COVID-19 pandemic and the post-COVID-19 era. [26] Physicians, trainers, researchers, healthcare providers, and decision makers in governmental and non-governmental healthcare systems should create guidelines to recommend adequate regular exercise in the post-COVID-19 era.

Our cross-sectional questionnaire survey had some limitations. The major limitation of this study was recall bias. Because we did not collect any data since the pandemic, the alternative was to ask for retrospective reports from the participants. Furthermore, it is possible that negative emotions such as fear or anger related to COVID-19 affected the results. Finally, this cross-sectional study could not evaluate HRQOL changes after the participants resumed their regular exercise regimens. Therefore, to overcome these limitations, a longitudinal questionnaire survey that had begun before the pandemic and includes a comprehensive sample of the general elderly population, would be helpful.

Meanwhile, the current survey has some strengths. The current survey was performed using self-administrated, printed questionnaires rather than online ones. This enables elderly people who might have difficulties participating in an online survey to participate in the survey and answer the questionnaire correctly based on their situation. Indeed, in the current study, we were able to obtain the responses of 95.1% of those asked to participate in the survey, and 14,494 out of 15,788 (96.5%) participants could complete the fundamental questionnaires. In addition, we were able to analyze the data of 45.3% of all elderly people living in the specific city, which significantly reduced the generalizability bias.

## Conclusion

The current population-based, cross-sectional survey that included 45.3% of elderly people living in a city revealed that up to 30% of participants were aware of the decrease in their regular exercise since the COVID-19 pandemic. This decrease was significantly related to HRQOL deterioration independent of age, sex, BMI, baseline ADL status, and musculoskeletal symptoms. Although our results might not be surprising for physicians, we believe that this knowledge could help the general population, healthcare providers, and decision-makers to understand the current problem and could be a solid foundation for creating exercise guidelines in the post-COVID-19 era.

## Data Availability

The datasets generated and/or analyzed during the current study are not publicly available due to privacy and ethical concerns but can be availed from the corresponding author upon reasonable request.

## Abbreviations

ADL: Activities of daily living
ANOVA: One-way analysis of variance
aORs: Adjusted odds ratios
BMI: Body mass index
COVID-19: Coronavirus disease
EQ-5D-5L: EuroQoL 5-dimension 5-level
HRQOL: Health-related quality of life
QOL: Quality of life

## References

[1] Zhu N, Zhang D, Wang W, Li X, Yang B, Song J, Zhao X, Huang B, Shi W, Lu R, Niu P, Zhan F, Ma X, Wang D, Xu W, Wu G, Gao GF, Tan W, China Novel Coronavirus I, Research T (2020). A Novel Coronavirus from Patients with Pneumonia in China, 2019. N Engl J Med.

[2] Terai H, Tamai K, Takahashi S, Hori Y, Iwamae M, Ohyama S, Yabu A, Hoshino M, Nakamura H (2021). The health-related quality of life of patients with musculoskeletal disorders after the COVID-19 pandemic. Int Orthop.

[3] Yamada M, Kimura Y, Ishiyama D, Otobe Y, Suzuki M, Koyama S, Kikuchi T, Kusumi H, Arai H (2020). Effect of the COVID-19 epidemic on physical activity in community-dwelling older adults in Japan: a cross-sectional online survey. J Nutr Health Aging.

[4] Chen P, Mao L, Nassis GP, Harmer P, Ainsworth BE, Li F (2020). Coronavirus disease (COVID-19): the need to maintain regular physical activity while taking precautions. J Sport Health Sci.

[5] Dwyer MJ, Pasini M, De Dominicis S, Righi E (2020). Physical activity: Benefits and challenges during the COVID-19 pandemic. Scand J Med Sci Sports.

[6] Garcia-Hermoso A, Ramirez-Velez R, Saez de Asteasu ML, Martinez-Velilla N, Zambom-Ferraresi F, Valenzuela PL, Lucia A, Izquierdo M (2020). Safety and effectiveness of long-term exercise interventions in older adults: a systematic review and meta-analysis of randomized controlled trials. Sports Med.

[7] Saint-Maurice PF, Troiano RP, Bassett DR, Graubard BI, Carlson SA, Shiroma EJ, Fulton JE, Matthews CE (2020). Association of daily step count and step intensity with mortality among US adults. JAMA.

[8] Rafferty MR, Schmidt PN, Luo ST, Li K, Marras C, Davis TL, Guttman M, Cubillos F, Simuni T, all NPF-QII Investigators (2017). Regular Exercise, Quality of Life, and Mobility in Parkinson's Disease: A Longitudinal Analysis of National Parkinson Foundation Quality Improvement Initiative Data. J Parkinsons Dis.

[9] Chen X, Zheng Y, Zheng W, Gu K, Chen Z, Lu W, Shu XO (2009). The effect of regular exercise on quality of life among breast cancer survivors. Am J Epidemiol.

[10] https://www.city.habikino.lg.jp/soshiki/soumu/soumu/jinko/jn_setai1.html. 2021. Habikino City official home page (in Japanese).

[11] Japanese Ministry of Health, L. a. W. Criteria for determination of the daily life independence level. https://www.mhlw.go.jp/english/database/db-hss/dl/siel-2010-04.pdf.

[12] Herdman M, Gudex C, Lloyd A, Janssen M, Kind P, Parkin D, Bonsel G, Badia X (2011). Development and preliminary testing of the new five-level version of EQ-5D (EQ-5D-5L). Qual Life Res.

[13] Shiroiwa T, Fukuda T, Ikeda S, Igarashi A, Noto S, Saito S, Shimozuma K (2016). Japanese population norms for preference-based measures: EQ-5D-3L, EQ-5D-5L, and SF-6D. Qual Life Res.

[14] Shiroiwa T, Ikeda S, Noto S, Igarashi A, Fukuda T, Saito S, Shimozuma K (2016). Comparison of Value Set Based on DCE and/or TTO Data: Scoring for EQ-5D-5L Health States in Japan. Value Health.

[15] Haberman SJ (1973). The Analysis of Residuals in Cross-Classified Tables. Biometrics.

[16] Coretti S, Ruggeri M, McNamee P (2014). The minimum clinically important difference for EQ-5D index: a critical review. Expert Rev Pharmacoecon Outcomes Res.

[17] Ekelund U, Steene-Johannessen J, Brown WJ, Fagerland MW, Owen N, Powell KE, Bauman A, Lee IM, Lancet Physical Activity Series 2 Executive C, Lancet Sedentary Behaviour Working G. Does physical activity attenuate, or even eliminate, the detrimental association of sitting time with mortality? A harmonised meta-analysis of data from more than 1 million men and women. Lancet. 2016;388(10051):1302–10. 10.1016/S0140-6736(16)30370-1 27475271

[18] Hupin D, Roche F, Gremeaux V, Chatard JC, Oriol M, Gaspoz JM, Barthelemy JC, Edouard P (2015). Even a low-dose of moderate-to-vigorous physical activity reduces mortality by 22% in adults aged >/=60 years: a systematic review and meta-analysis. Br J Sports Med.

[19] Kyu HH, Bachman VF, Alexander LT, Mumford JE, Afshin A, Estep K, Veerman JL, Delwiche K, Iannarone ML, Moyer ML, Cercy K, Vos T, Murray CJ, Forouzanfar MH (2016). Physical activity and risk of breast cancer, colon cancer, diabetes, ischemic heart disease, and ischemic stroke events: systematic review and dose-response meta-analysis for the Global Burden of Disease Study 2013. BMJ.

[20] Colberg SR, Sigal RJ, Yardley JE, Riddell MC, Dunstan DW, Dempsey PC, Horton ES, Castorino K, Tate DF (2016). Physical Activity/Exercise and Diabetes: A Position Statement of the American Diabetes Association. Diabetes Care.

[21] Piercy KL, Troiano RP, Ballard RM, Carlson SA, Fulton JE, Galuska DA, George SM, Olson RD (2018). The Physical Activity Guidelines for Americans. JAMA.

[22] Algahtani FD, Hassan SU, Alsaif B, Zrieq R (2021). Assessment of the Quality of Life during COVID-19 Pandemic: A Cross-Sectional Survey from the Kingdom of Saudi Arabia. Int J Environ Res Public Health.

[23] Paluska SA, Schwenk TL (2000). Physical activity and mental health: current concepts. Sports Med.

[24] Geneen LJ, Moore RA, Clarke C, Martin D, Colvin LA, Smith BH (2017). Physical activity and exercise for chronic pain in adults: an overview of Cochrane Reviews. Cochrane Database Syst Rev.

[25] Costanzo G, Misaggi B, Ricciardi L, AlEissa SI, Tamai K, Alhelal F, Alqahtani Y, Alsobayel HI, Arand M, Balsano M, Blattert TR, Brayda-Bruno M, Busari JO, Campello M, Chhabra HS, Tamburrelli FC, Cote P, Darwono B, Kandziora F, La Maida GA, Muehlbauer EJ, Mulukutla RD, Pereira P, Rajasekaran S, Rothenfluh DA, Sullivan WJ, Truumees E, Dohring EJ, Pigott T, Shetty AP, Teli MGA, Wang JC, Ames C, Anema JR, Bang A, Cheung KMC, Gross DP, Haldeman S, Minisola S, Mullerpatan R, Negrini S, Salmi LR, Spinelli MS, Vlok A, Yankey KP, Zaina F, Alturkistany A, Franke J, Liljenqvist UR, Piccirillo M, Nordin M (2022). SPINE20 recommendations 2021: spine care for people's health and prosperity. Eur Spine J.

[26] Galea S, Vaughan R (2021). Preparing the Public Health Workforce for the Post-COVID-19 Era. Am J Public Health.

